# Prevalence of Dens Invaginatus assessed by CBCT: Systematic Review and Meta-Analysis

**DOI:** 10.4317/jced.59849

**Published:** 2022-11-01

**Authors:** Sara González-Mancilla, Paloma Montero-Miralles, Juan J. Saúco-Márquez, Victoria Areal-Quecuty, Daniel Cabanillas-Balsera, Juan J. Segura-Egea

**Affiliations:** 1 DDS, Department of Stomatology, Section of Endodontics, School of Dentistry, University of Sevilla, C/ Avicena s/n, 41009-Sevilla, Spain; 2 DDS, MSc, PhD, Professor of Master in Clinical Endodontics, University of Sevilla, C/ Avicena s/n, 41009-Sevilla, Spain; 3 MD, DDS, PhD, Professor of Master in Clinical Endodontics, University of Sevilla, C/ Avicena s/n, 41009-Sevilla, Spain; 4 DDS, MSc, Doctoral fellow, University of Sevilla, C/ Avicena s/n, 41009-Sevilla, Spain; 5 MD, DDS, PhD, Professor, Department of Stomatology, Section of Endodontics, School of Dentistry, University of Sevilla, C/ Avicena s/n, 41009-Sevilla, Spain

## Abstract

**Background:**

Dens invaginatus is a developmental dental anomaly resulting from an invagination of dental tissues folding from the outer surface towards dental pulp. The aim of this systematic review and meta-analysis was to determine the prevalence of dens invaginatus using cone beam computed tomography (CBCT).

**Material and Methods:**

A systematic review was conducted following PRISMA statements. The research question was: What is the prevalence of dens invaginatus in the adult population assessed by CBCT? The MeSH terms were used to search articles published in the electronic database PubMed. Studies were selected considering predetermined eligibility criteria. The Robins-I tool developed by Cochrane was used to assess methodological quality and risk of bias.

**Results:**

Four studies were included in this systematic review, including 2009 CBCT images. The overall prevalence of dens invaginatus was 9.0% (95% CI = 7.2 – 10.8%; *p*< 0.001). Three studies were considered of low risk of bias.

**Conclusions:**

The results of this systematic review and meta-analysis show that prevalence of dens invaginatus using CBCT was higher than previous estimations carried out with conventional radiographs. Therefore, an early identification and a correct management of invaginated teeth is essential for improving the prognosis of these teeth. It can be concluded that teeth with dens invaginatus should always be studied using CBCT.

** Key words:**Dens invaginatus, Dens in dente, Dental anomalies, CBCT, Cone beam computed tomography.

## Introduction

Dens invaginatus, also known as dens in dente, is a dental development anomaly that results in an invagination into the tooth, because dental tissues creates a folding from the outer surface towards the pulp, prior to dental tissues calcification (1).

Many etiopathogenic theories have been suggested to explain this process. Kronfeld *et al*. (2) suggested that it was result of a retarded proliferation of a particular group of cells, while the surrounding cells continue proliferating normally. Rushton (3) suggest that the cause of this anomaly was embryological, due to the stimulation and subsequent proliferation and growth of enamel organ cells into the papilla during tooth development. Atkinson (4) suggested a mechanical cause, due to external forces that have an effect on the tooth germ during development. These forces could came from adjacent tooth germs, for example central incisors or canines, which develop is at least 6 months before lateral incisors and could press the lateral incisor germ (5). Other factors such as trauma (6) and infection have also been proposed as causes of this anomaly (7).

Dens invaginatus can occur in both dentitions, with a prevalence ranging from 0.25% to 7.7% (8), although it is more common in permanent dentition. There have been described cases of maxillary central and lateral incisors, canines and premolars, as well as mandibular incisors and premolars (9), being the maxillary lateral incisors the most frequently involved, sometimes bilaterally, and the occurrence of dens invaginatus in supernumerary teeth is also frequent (10). Also, dens invaginatus has been found in several members of the same family (11), showing a genetic component.

Depending on the location and how was the tooth affected, two types of invaginations are distinguished: coronal and radicular invaginations. The classification proposed by Oehlers in 1957 (12) allows us to highlight three types of invaginations, according to their radiographic extension from crown to root:

• Type I: is a minimal invagination, enamel-lined and confined within the crown of the tooth. It is the most common lesion, with a frequency of 79%.

• Type II: the invagination extends apically to the amelocemental line. It forms a blind dead-end that may or not communicate with the pulp but remains within the root canal without communication with periodontal ligament.

• Type III: the invagination extends through the root. Normally there is no pulp communication, which is compressed within the root. Two sub-types can be stablished: type IIIa, when communicates laterally with periodontal space through a pseudo-foramen, and type IIIb, when the invagination extends through the root and communicates with the periodontal ligament in the apical foramen.

On the other hand, Cone Beam Computed Tomography (CBCT) is a radiographic method that provides a three-dimensional image of the entire canal system, overcoming some of limitations of conventional periapical radiography, such as distortion or overlapping of structures (13). Different studies have shown that CBCT reveals more endodontic radiographic findings than conventional imaging methods (14,15). However, its use in endodontics is not routinely indicated, and it should be used only when the examination demonstrate that benefits to the patient exceed outweigh potential risks. The ALARA principle (“as low as reasonably achievable”) had to be considered in all cases (16).

According to the European Society of Endodontology (ESE) and the American Association of Endodontists (AAE), CBCT application in endodontics is recommended in situations as diagnosis, surgical and non-surgical endodontic retreatment, resorptions and dentoalveolar traumatology (16).

The aim of this study was to conduct a systematic review and meta-analysis of the prevalence of dens invaginatus in adults using CBCT.

## Material and Methods

This study did not required ethical approval, since used data was freely available in the public domain.

-Research question

The review question was formulated following the CoCoPop mnemonic (17), as follows: What is the prevalence of dens invaginatus in adult population assessed by CBCT?

-Inclusion and exclusion criteria

Inclusion criteria were as follows: studies in adults where diagnosis of dens invaginatus was carried out using CBCT. In addition, exclusion criteria were: studies with diagnosis of dens invaginatus using conventional radiography, studies that do not determine prevalence of dens invaginatus and case reports.

-Literature search strategy

This study was conducted following the PRISMA statements (18). Searching process was carried out by two reviewers (J.J. S-E and S.G-M), with a search for articles in PubMed and SCOPUS electronic databases until 21 October 2021, with no restrictions or limits on language or year.

The following combination of Medical Subject Heading (MeSH) terms and keywords were used: (dens invaginatus OR dens in dente) AND (prevalence or frequency) AND (cone OR CBCT OR tomography).

-Data extraction

Two examiners (J.J.S-E and S.G-M) compiled the data of included studies. The following data were extracted: author and year of publication, type of study, CBCT machine and acquisition parameters, number of men and women included, number of evaluated teeth, prevalence of dens invaginatus, distribution according sex, type of tooth, type of dens invaginatus (according to Oehler’s classification), unilateral or bilateral presence, and main results.

Meta-analysis: outcome variables and statistical analysis

The outcome variable was the prevalence of dens invaginatus, calculated with a 95% confidence interval (CI). Meta-analysis on the included studies was carried out using OpenMeta Analyst software. To estimate variance and heterogeneity of the studies, Tau2 and Higgins I2 tests were used, considering a low heterogeneity if it was less than 25%, slight between 25 and 50%, moderate between 50 and 75% and high if was greater than 75% (19). A statistical significance was considered when *p-value* was < 0.05.

-Quality assessment and risk of bias

To establish the level of evidence for each included studie, the Oxford scale of evidence has been used (20). In addition, to assess the risk of bias the Risk of Bias Tool for prevalence studies developed by Hoy *et al*. (21) was used. This tool comprises 10 items plus a summary assessment. Items 1 to 4 assess the external validity of the study (domains are selection and nonresponse bias), and items 5 to 10 assess the internal validity (items 5 to 9 assess the domain of measurement bias, and item 10 assesses bias related to the analysis).

## Results

-Searching strategy

**Figure 1:**
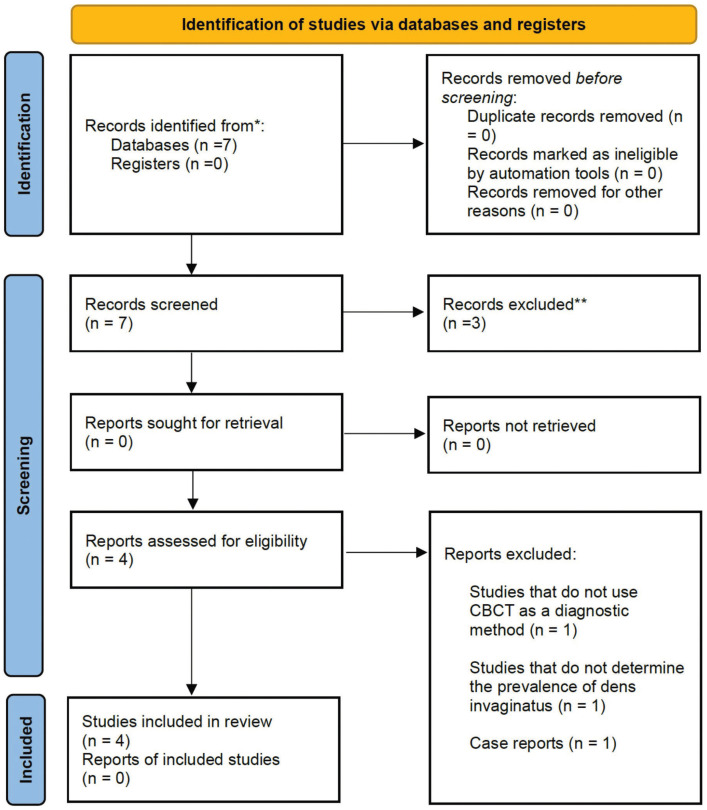
PRISMA flowchart for systematic literature review and article inclusion.

The searching strategy flowchart is shown in Fig. 1. Seven articles were identified after searching the PubMed database. In the screening, there were no duplicate papers. After reviewing the full-text papers, four papers met the inclusion criteria, as three were excluded for the following reasons: one did not use CBCT diagnosis, another didn´t assess prevalence of dens invaginatus, and another assesses internal anatomy of maxillary incisors by CBCT according to Vertucci’s classification.

Included studies and their essential characteristics 

Finally, four studies were included for the systematic review and meta-analysis: (22-25), which characteristics are shown in Table 1.

**Table 1:**
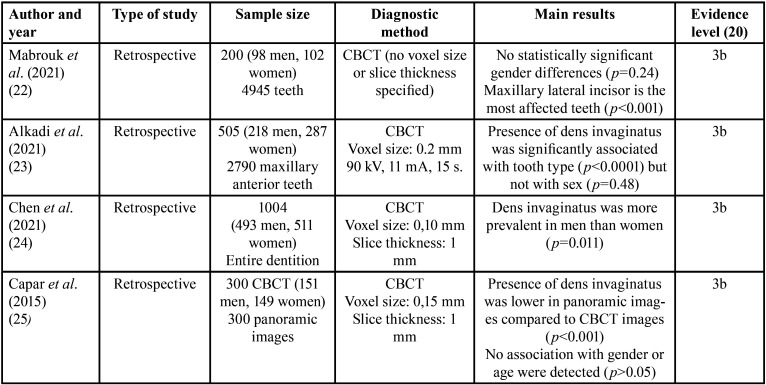
Main characteristics and evidence level of the studies included in the systematic review.

Three of the included studies are limited to analyze dens invaginatus prevalence by CBCT imaging, without comparison with other radiographic diagnosis methods. However, the article by Capar *et al*. (25) is a comparative study of CBCT versus rendered panoramic images in determining the prevalence of this dental anomaly.

Studies by Alkadi *et al*. (23), Chen *et al*. (24), and Mabrouk *et al*. (22) conclude that CBCT provides an accurate representation of both external and internal dental anatomy, helping in the diagnosis of dens invaginatus. Furthermore, they refer the study by Capar *et al*. (25), which shows that detection of dens invaginatus was lower in panoramic images (3%) compared to CBCT images (10.7%).

-Meta-analysis

Data from selected articles were analysed and summarised in Table 2. To carry out the meta-analysis, the results of three of the included studies (22-24) were incorporated in full because these studies used only CBCT to diagnose dens invaginatus. On the contrary, from the study of Capar *et al*. (25), only data regarding the use of CBCT were employed. In total, the results of the four included studies compiled 2009 CBCT images.

**Table 2:**
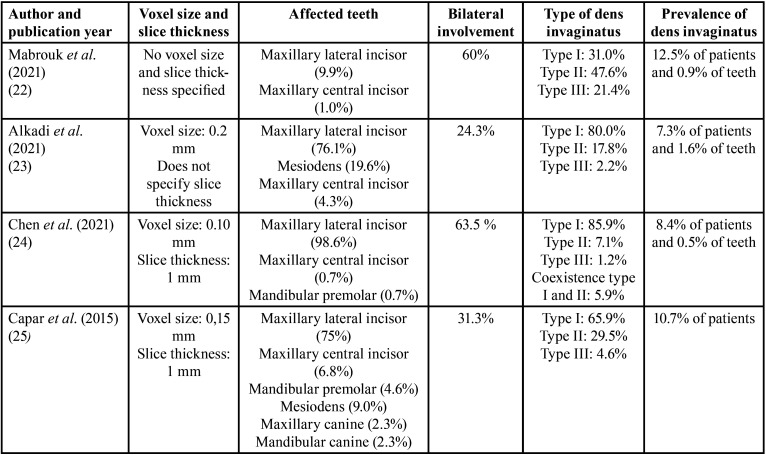
Extracted and compiled data: affected teeth, bilateral involvement, type of dens invaginatus, prevalence of dens invaginatus and descriptive statistics.

The estimated variance between all results was examined by Tau2 test, resulting not significant (Tau2 = 0.00; df = 5.332; *p* = 0.149). In this case, the value of Tau2 being 0 indicates that there are no additional values affecting weighting of studies. The heterogeneity test value (I2 = 43.73%) gave a result that represents slight heterogeneity. Furthermore, it should be noted that the weights of studies were calculated using the random effects model considering that there was variation between included studies and allowing the results to vary in a normal distribution. The overall proportion or prevalence of dens invaginatus was 0.090 (95% CI = 0.072 – 0.108; *p* < 0.001), indicating a significant influence of CBCT diagnosis in determining the prevalence of dens invaginatus.

**Figure 2:**
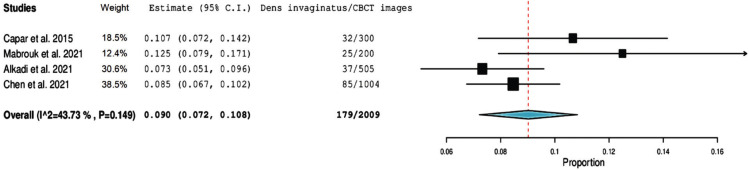
Forest plot of systematic review and meta-analysis results.

Prevalence of dens invaginatus from each of included studies and the pooled prevalence calculated from the meta-analysis were shown in a forest plot (Fig. 2).

-Interpretation and assessment of each of the included studies

The four studies included in the meta-analysis were retrospective, and data obtained from studies were compiled: 179 dens invaginatus diagnosed in 2009 CBCT images.

The overall result of meta-analysis indicates a prevalence of dens invaginatus of 0.090 (95% CI = 0.072 – 0.108; *p* < 0.001), indicating a significant influence of CBCT diagnosis in determining the prevalence of dens invaginatus. It is a significantly higher result compared to the previously known prevalence of dens invaginatus using conventional radiography as a diagnostic method.

Study by Chen *et al*. (24) uses CBCT images of 1004 patients (493 men and 511 women) from 17 to 73 years in a Chinese population. Dens invaginatus was observed in 85 of 1004 patients (prevalence 8.47%), being more prevalence in males than females (6.68% females and 10.75% males).

Study by Alkadi *et al*. (23) uses CBCT images of 505 patients (218 men and 287 women), from 8 to 70 years in a Saudi population. To assess the prevalence of dens invaginatus, 2790 maxillary anterior teeth were evaluated, detecting dens invaginatus in 37 of 505 patients (prevalence 7.3%). No significant differences were found regarding to gender.

Study by Mabrouk *et al*. (22) uses CBCT images of 200 patients (98 men and 102 women) from 16 to 89 years in a Tunisian population, evaluated 4945 maxillary and mandibular anterior teeth to study the prevalence, characteristics and type of dens invaginatus. Results showed that 25 of 200 patients (prevalence 12.5%) had dens invaginatus. Men showed more prevalence,but with no statistically significant differences.

Finally, Study by Capar *et al*. (25) uses CBCT images of 300 patients (151 men and 149 women) from 8 to 71 years in a Turkish population, to determine the presence, characteristics and classification of the type of dens invaginatus. In addition, 300 panoramic images were used to compare the results. Based on CBCT images, dens invaginatus was observed in 32 of 300 patients (prevalence 10.7%), with no significant gender differences. On the other hand, based on panoramic images, dens invaginatus was observed in 9 of 300 patients (prevalence 3%).

All studies found this anomaly with more frequency in the upper lateral incisor, followed by the upper central incisor, and Type I according to Oehler’s classification is the most frequent form of presentation.

Quality assessment and risk of bias

The score obtained to establish the level of evidence for each included studies, according to the Oxford evidence scale (20), was moderate (Table 1), with all studies scoring 3b as they were retrospective studies.

**Figure 3:**
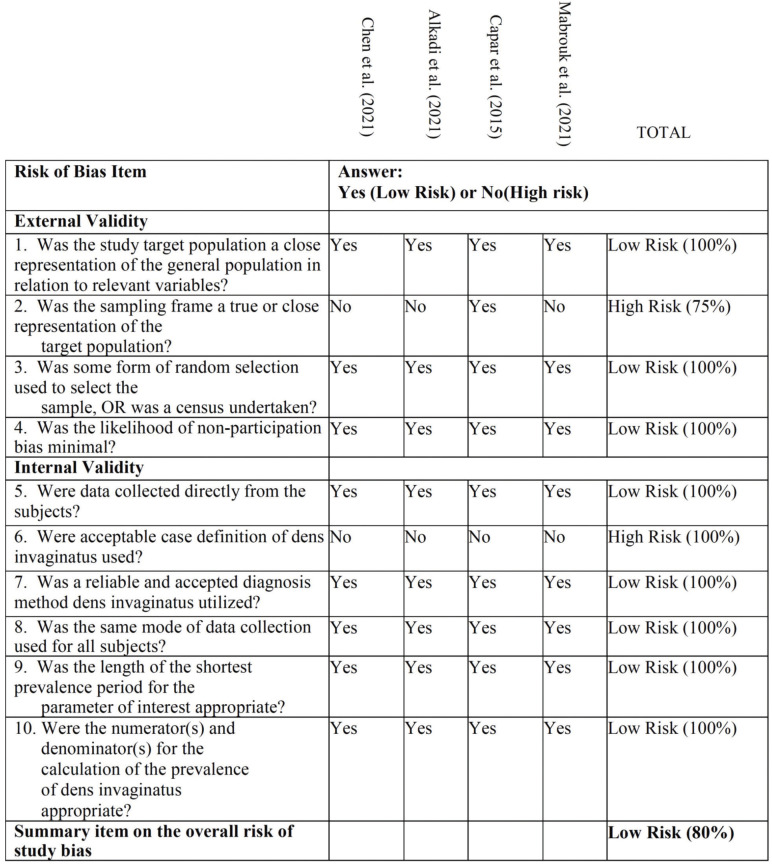
Quality assessment and risk of bias, assessed using the Risk of Bias Tool for Prevalence Studies (21).

On the other hand, the methodological quality and risk of bias was assessed using the Risk of Bias Tool for Prevalence Studies, developed by Ho *et al*. (21). The total percentage of reported parameters was 80%, indicating a low overall risk of bias (Fig. 3).

## Discussion

In this systematic review and meta-analysis, the prevalence of dens invaginatus has been analyzed using CBCT as a diagnostic method. Results show that prevalence of dens invaginatus obtained using CBCT as a diagnostic method is significantly higher (9%) than data obtained by using two-dimensional imaging (0.25% to 7.7%) (8). Therefore, CBCT is an effective and essential tool for accurately diagnose and treatment of this dental anomaly. These results are in accordance with those of Capar *et al*. (25), where was highlighted the difference in prevalence using CBCT (10.7%) compared to conventional radiography (3%).

To our knowledge, this is the first systematic review analyzing the prevalence of dens invaginatus using CBCT for diagnosis, a topic that has not been investigated so far in meta-analyses. Thus, results of the present study should be considered to reaffirm that CBCT does indeed help to fulfil the diagnosis of dens invaginatus, providing an accurate representation of the dental anatomy.

Conventional radiographic techniques have numerous limitations, including anatomical noise, various degrees of geometric distortion as well as the two-dimensional nature of the images obtained. In addition, interpretation of these images may also be incorrect due to the anatomical characteristics of each region and the overlapping of other adjacent teeth and dentoalveolar structures (13). In contrast, CBCT largely overcomes these limitations, and provides a lower spatial resolution than periapical radiographs, showing structures in all three dimensions of space (26), althought it also showed disadvantages as a decrease in image quality (27) particularly in presence of highly radiopaque objects, such as metal restorations, posts and guttapercha. This has led to an increasing use of CBCT in endodontics (28), and many professional organizations recommend it (16). Nevertheless, benefits must outweigh the higher levels of radiation exposure compared to conventional imaging (29).

As can be seen, CBCT is not a perfect radiographic diagnostic method, which would also be utopian, but despite its limitations, it far surpasses the two-dimensional radiography used until the advent of CBCT as the only radiographic method. In addition, its numerous indications include the precise and accurate assessment of anatomically complex root canal systems for endodontic treatment, such as dens invaginatus (26).

It is noteworthy that three of the four included studies specify the voxel size used in the CBCT, which allows for a homogeneous comparison of the results. Voxel is the smallest 3D element of an acquired volume and is usually represented as a cube or box with a given height, width, and depth. Just as a 2D image consists of several pixels, and the smaller the pixels are, the higher the image quality is, the same concept applies to the volume of data acquired by CBCT. Small voxel sizes (Table 2) have been found, indicating higher image resolution and greater ability to differentiate small structures.

With regard to the limitations of the present review, only four studies were found to met the inclusion criteria, which is a rather low number, although the quality of all of them is acceptable with a low to moderate risk of bias. The study by Chen *et al*. (24) studied and reported results from a sample of the Beijing population and study by Alkadi *et al*. (23), which is based on a convenience sample that may not be representative of the population. In addition, large-scale multi-centre studies are needed.

## Conclusions

The prevalence of dens invaginatus assessed using CBCT is significantly higher than data obtained by using two-dimensional imaging. CBCT is a key diagnostic method for identification of dens invaginatus, as well as for determining its prevalence. Therefore, considering that early identification and management of dens invaginatus is critical to improve the affected teeth prognosis, this anomaly, or any suspicion of it, should always be investigated by CBCT, as it provides a better definition and accuracy.
